# Translation of Plant RNA Viruses

**DOI:** 10.3390/v13122499

**Published:** 2021-12-13

**Authors:** Guowei Geng, Deya Wang, Zhifei Liu, Yalan Wang, Mingjing Zhu, Xinran Cao, Chengming Yu, Xuefeng Yuan

**Affiliations:** 1 Shandong Province Key Laboratory of Agricultural Microbiology, Department of Plant Pathology, College of Plant Protection, Shandong Agricultural University, Tai’an 271018, China; guowgeng@163.com (G.G.); wliuzhifei@163.com (Z.L.); wangyalan97@163.com (Y.W.); zhumingjing98@163.com (M.Z.); 2 Department of Biotechnology, College of Life Sciences, Zaozhuang University, Zaozhuang 277160, China; wangdeyasdny@163.com; 3 Department of Plant Pathology, College of Plant Health and Medicine, Qingdao Agricultural University, Qingdao 266109, China; xinran1001@163.com

**Keywords:** plant RNA viruses, cap-independent translation, translation recoding

## Abstract

Plant RNA viruses encode essential viral proteins that depend on the host translation machinery for their expression. However, genomic RNAs of most plant RNA viruses lack the classical characteristics of eukaryotic cellular mRNAs, such as mono-cistron, 5′ cap structure, and 3′ polyadenylation. To adapt and utilize the eukaryotic translation machinery, plant RNA viruses have evolved a variety of translation strategies such as cap-independent translation, translation recoding on initiation and termination sites, and post-translation processes. This review focuses on advances in cap-independent translation and translation recoding in plant viruses.

## 1. Introduction

Plant viruses usually encode several viral proteins essential for the important processes in the viral life cycle, such as replication, translation, movement, and virus packaging. However, translation of viral proteins totally depends on the host translation machinery, which prefers RNA templates with the same characteristics as cellular mRNA. For DNA viruses, the genomic transcripts have the same characteristics as cellular mRNA since their viral genome enters the nucleus. Translation of the viral RNA for DNA viruses is accomplished using the canonical ribosome scanning model. For RNA viruses, their viral genome does not enter the nucleus and remains the intrinsic characteristic of the virus particle. The genomic RNA of many plant RNA viruses lacks the 5′ cap and/or 3′ poly(A) as cellular mRNA, which are essential factors ensuring the effective translation of cellular mRNA. In addition, the viral RNA of some RNA viruses is multi-cistronic, which is remarkably different from mono-cistronic cellular mRNA. It is suggested that the internal open reading frame (ORF) and ORFs located at the 3’ part may be expressed by several strategy. Although most plant RNA viruses present differences in terms of the 5′ end, 3′ end, and encoding characteristic from cellular mRNA, they accomplish the expression of viral proteins through many strategies, including the synthesis of subgenomic RNA at the transcriptional level, cap-independent translation, and translation recoding at the translational level [1,2,3]. This review focuses on advances in cap-independent translation and translation recoding in plant viruses.

## 2. Characteristics of 5′ and 3′ Ends of Viral RNAs in Plant RNA Viruses

To study the translation of viral proteins, the characteristics of the 5′ and 3′ ends of the viral RNA are the primary factors to be considered. If viral RNA has a capped 5′ end and polyadenylated 3′ end, it can express the viral protein through the canonical ribosome scanning model as cellular mRNA. In the ribosome scanning model of eukaryotic cellular mRNA, the 7-methylguanosine cap at the 5′ end of cellular mRNA is first bound by eIF4E, part of the eIF4F complex, which recruits the 43S preinitiation complex, including the 40S small ribosomal subunit, eIF2–GTP–Met-tRNAi ternary complex, along with eIF1, eIF1A, eIF3, and eIF5. The resulting 48S preinitiation complex is then scanned from 5′ to 3′ in an ATP-dependent manner until the charged initiator Met-tRNAi is base-paired with an AUG start codon surrounding by suitable context sequences. A number of initiation factors are then displaced to allow the joining of the 60S large ribosomal subunit to form the complete 80S ribosome to initiate translation. The 3′ poly (A) can be bound by PABP, which also binds eIF4G to cyclize the cellular mRNA, greatly enhancing translation by avoiding cellular mRNA decay and cyclic utilization of ribosomal subunits [4,5].

Here, all genera of plant RNA viruses are listed with characteristics of the 5′ and 3′ ends, as well as the potential translation recoding strategy (Table 1). Of the 106 assigned genera of plant RNA viruses [6,7], only 18 (17.0%) had genomic RNA with both 5′ cap and 3′ poly(A), while 24 (22.6%) had genomic RNA without 5′ cap and 3′ poly(A) (Table 1). In addition, 64 (60.4%) had genomic RNA with a 5′ cap (30 genera) or 3′ poly(A) (34 genera) (Table 1). For genera without the 5′ cap, seven in the family *Secoviridae*, two in the family *Solemoviridae*, 12 in the family *Potyviridae* of the order *Patatavirales,* and three in the family *Luteoviridae* had a viral genome-linked protein (VPg) at the 5′ end of the genomic RNA. For genera without 3′ poly(A), six in the family *Bromoviridae*, seven in the family *Virgaviridae,* and one in the family *Tymoviridae* had a 3′ tRNA-like structure (Table 1). There were 58 genera (54.7%) of plant RNA viruses that lack the 5′ cap (Table 1), which may translate the viral proteins through a cap-independent translation strategy to recruit translation factors in different manners.

## 3. Cap-Independent Translation in Plant RNA Viruses

Cap-independent translation in plant RNA viruses is often mediated by two types of *cis*-elements: internal ribosome entry site (IRES) and 3′ cap-independent translation enhancer (3′ CITE). IRES was first reported in animal RNA viruses such as poliovirus and Encephalomyocarditis virus (EMCV), and it is mainly located at the 5′ upstream region of corresponding open reading frames (ORFs) [102,103]. 3′CITE was first reported in plant RNA viruses, such as satellite tobacco necrosis virus (sTNV), and it is located at 3′ downstream of the corresponding ORFs [2,104,105]. In addition to RNA viruses, IRES and 3′CITE were reported in eukaryotic cellular mRNAs, which may play a role when cap recognition is suppressed under special conditions and act as modulators of enhanced stress resistance, metabolic processes, and development [105,106,107,108].

### 3.1. IRESes in Plant RNA Viruses

Since IRES was first reported in picornavirus RNAs [102,103], it has been reported in many animal and plant RNA viruses, as well as in host cellular mRNAs [109,110,111,112,113]. For animal RNA viruses, viral IRESes are classified into six classes on the basis of their structural characteristics and the requirements for various translation initiation factors and IRES *trans*-acting factors (ITAFs). Picornavirus IRESes are classified into five types (I, II, III, HCV-like, and AV-like), and dicistrovirus IRESes are classified into another type [114,115,116,117]. The IRES sequences in animal RNA viruses are long (at least 450 nt), and the corresponding RNA tertiary structures are very complex. Various translation initiation factors, as well as ITAFs, are required by these IRESes to play a role during translation initiation [117,118,119,120,121,122,123,124]. There seemed to be an inverse correlation between the degree of the stable structure of the IRES element and the number of factors. Most animal IRESes do not require eIF4E, which is one of the main targets of cellular translation regulation [125]. IRES activity in some animal RNA viruses is also synergistically enhanced by the long-distance RNA–RNA interaction between the 5’ and 3’ ends [126,127,128,129,130,131,132].

Studies on IRESes in plant RNA viruses have mainly focused on members of the family *Potyviridae*, such as tobacco etch virus (TEV), turnip mosaic virus (TuMV), potato virus Y (PVY), triticum mosaic virus (TriMV), and wheat yellow mosaic virus (WYMV) [111,113,133,134,135,136]. IRESes in plant RNA viruses have shorter sequence lengths (60–190 nt, excluding TriMV) and simpler structures than those in animal RNA viruses. The characteristics of genomic RNA in the family *Potyviridae* are similar to those in the family *Picornaviridae*. They all have VPg at the 5’ end and poly (A) at the 3’ end. They all encode a single polyprotein to produce functional proteins via the cleavage of proteinases. However, the 5’UTR of the family *Potyviridae* is relatively shorter than that of the family *Picornaviridae* (about 150 nt in *Potyviridae* and 600–1200 nt in *Picornaviridae*), and the structure of the 5’UTR in the family *Potyviridae* is simpler than that in the family *Picornaviridae*. In addition, the VPg of the family *Potyviridae* is relatively larger than that in the family *Picornaviridae* (20–23 kDa in *Potyviridae*, and 2–3 kDa in *Picornaviridae*) [111]. VPg can interact with eIF4E or eIFiso4E, which was associated with the regulation on translation of viral RNA and host mRNA in several potyviruses such as TEV, TuMv and PVA [137,138,139,140]. Eukaryotic eIF4E-mediated recessive resistance to plant viruses were reported, which is a direction of virus-resistant crop breeding [141]. In the genus *Calicivirus*, VPg- eIF4E interaction is required for translation [142]. However, But the VPg of members in the family *Picornaviridae* is dispensable in the process of translation regulation [143]. TheVPg in the family *Picornaviridae* can be released from viral RNA though “unlinkase” activity via TDP2 enzyme [144]. Due to the two state of VPg including dissociative state and RNA-linked state, the relationship between VPg and translation is complicated. The possible function and mechanism of VPg in translation are not discussed here. Here, reported IRESes in plant RNA viruses are shown and summarized below.

#### 3.1.1. IRESes in Members of Family Potyviridae

TEV is a model virus used to study the translation regulation of the family *Potyviridae* [145,146]. Early studies have shown that two regulatory elements (CIRE and CIRE-2) in the 5’UTR (144 nt) of TEV can improve the cap-independent translation efficiency by 8–21-fold [133,147], and poly (A) can synergistically improve the translation efficiency mediated by the 5’UTR [147,148]. The 5’ proximal domain (38–75 nt) in TEV can fold into a pseudoknot (Ψ), which is essential for cap-independent translation [149]. In addition, loop sequences (UACUUCU) in L3 can pair with the 1117–1123 nt of 18S rRNA. It is suggested that the base-pairing sequences between the 5′UTR and 18S RNA may directly recruit ribosomal subunits to enhance translation [149]. When the 5’UTR of TEV is placed in the intergenic region of the bi-cistronic reporter vector, it promotes the expression of the second ORF, indicating IRES activity [150]. When a stable secondary structure is placed before the 5’ end, the IRES efficiency is reduced by nearly 10-fold, indicating that maximal activity of the IRES requires an open 5’ end. [147]. The IRES activity from the 5’UTR of TEV is eIF4F-dependent rather than eIFiso4F-dependent due to the interaction between eIF4G and the 5’UTR of TEV, and this interaction facilitates cap-independent translation [138,150,151].

Insertion of the 5’UTR (184 nt) of PVY into the intergenic region of the bi-cistronic reporter vector can promote the expression of the second gene, which indicates that the 5’UTR of PVY has IRES activity [134]. On the basis of this prediction, the 5’UTR of PVY has two hairpins [152]. Deletion of the first hairpin increases translation, whereas deletion of the second hairpin slightly decreases translation [152]. It is suggested that the first hairpin has a negative effect on IRES activity because of the potential block on the scanning of ribosomes [152]. In addition, the 3’ terminal 55 nt region in the 5’UTR of PVY is crucial for cap-independent translation [152]. However, the detailed mechanism and required translation factors have not yet been determined.

The 5’UTR of TuMV (130 nt) can promote translation in vivo and in vitro, but the exact mechanism is still unclear [135]. When a stable hairpin is added before the TuMV 5’UTR, its translation level is reduced by 70%. The complementary sequences to the 5’UTR of TuMV inhibited cap-independent translation in a *trans* competition experiment, while the identical complementary sequences located at reporter gene increased translation [135]. It is suggested that both 5’UTR of TuMV and its complementary sequences can support the cap-independent translation.

TriMV is a newly discovered virus that infects wheat. Compared with other members of the genus *Potyvirus*, its 5’UTR with 739 nt is very long and its opening reading frame starts from the 13th initiation codon [153,154]. The 5’UTR of TriMV enhances cap-independent translation in vivo and in vitro [136]. IRES activity from the TriMV 5’UTR requires a hairpin structure at position 469–490 nt [136]. The 5’UTR of TriMV can directly interact with eIF4G and eIFiso4G, and the hairpin structure at position 469–490 nt is very important for this interaction between the 5’UTR and eIF4G [155]. In addition, cap-independent translation mediated by the 5’UTR also requires eIF4A instead of eIF4E [155]. 

In PVA, another member of the genus *Potyvirus*, the 5’UTR (161 nt) without remarkable structure characteristic played a key role in the translation of viral RNA stimulated by VPg and ribosomal protein P0 [156]. It is implied the possible synergistic function of VPg and 5’UTR on translation. 

Recently, a novel IRES element was found in the 5′UTR (162 nt) of RNA1 of WYMV [103]. The core elements of IRES in WYMV RNA1 have two hairpins (H1 and H2) and an internal linker region (LR1). IRES activity from the 5′UTR can be synergistically enhanced via long-distance RNA–RNA interaction between C^80^U in the 5′UTR and A^7574^G in the 3′UTR [103]. Structural stability of the stem and nucleotide specificity of the upper loop in H1, along with the length of discontinuous stems and nucleotide specificity of the upper loop in H2 are the core *cis*-element for IRES activity from the 5′UTR [103]. The IRES of WYMV RNA1 5′UTR is eIF4E-dependent, and the target site of eIF4E is the top loop of H2, especially C^114^UUUCC [103]. In addition, the cytosines (C^55^, C^66^, C^105^, and C^108^) in the hairpins H1 and H2 and the guanines (G^73^, G^79^, and G^85^) in LR1 form discontinuous base pairing to maintain a dynamic equilibrium state. Dynamic base pairs between C^55^ and C^66^ in H1 and guanines (G^73^, G^79^, and G^85^) in LR1 have positive effects on IRES activity, while dynamic base pairs between C^105^ and C^108^ in H2 and guanines (G^73^, G^79^, and G^85^) in LR1 negatively regulate the IRES activity [103]. Dynamic base pairs among cytosines (C^55^, C^66^, C^105^, and C^108^) in H1/H2 and guanines (G^73^, G^79^, and G^85^) in LR1 maintain a tertiary equilibrium state to ensure that the IRES activity of the RNA1 5’UTR is at a suitable level, which is suggested to be the evolution target of WYMV RNA1 [103].

#### 3.1.2. IRESes in Other Plant RNA Viruses

In addition to IRES found in species of the family *Potyviridae*, it has been reported in other types of plant RNA viruses. The upstream coat protein (CP) ORF in TCV contains an IRES, which can regulate the expression of CP protein [157]. The low-level expression of CP protein can be detected even if CP subgenomic RNA is not synthesized. The IRES located upstream of the CP ORF in TCV does not present structural characteristics, and the IRES activity is related to an unstructured A-rich sequence. Moreover, IRES activity depends on eIF4G instead of eIF4E [157]. IRES with A-rich sequences has also been found upstream of the CP and MP ORFs in crTMV [158]. Similar IRES elements have also been found in a variety of viruses of the family *Tombusviridae*, such as HCRSV and PFBV [159,160]. These IRES elements present unstructured characteristic [157,158,159,160]. An IRES has been found in the 5’UTR of RNA2 of the blackcurrant conversion virus (BRV), a *Nepovirus*. This IRES did not present a remarkable secondary structure, but it did contain multiple segments of an 8–10 nt sequence motif essential for IRES activity, which can complement the position of the 1113–1123 nt region of 18S rRNA [161]. Subsequently, similar regions complementary to 18S rRNA have been found in the 5’UTR of other species of *Nepovirus*. It is speculated that these IRESes may directly recruit 40S subunit, because 18s rRNA is part of the 40S subunit [161]. IRES has also been found in the genome of the potato leaf roll virus (PLRV). The IRES is completely located in the ORF. The core *cis*-elements include a conserved AUG codon and adjacent inverse symmetric motif (GGAGAGAGAGG) [162].

To date, IRESes of plant RNA viruses have presented multifarious structural characteristics and can be roughly divided into three types according to their structural characteristics (Table 2). Type I IRES is unstructured and generally contains a section of an A-rich sequence. The representative viruses are TCV/HCRSV/PFBV/BRV. Type II IRES is structural. All of these IRESes have one or several hairpins. According to the numbers and other characteristics of hairpin structures, they can be divided into the single hairpin type such as in TEV, the double hairpin type such as in PVY and TriMV, and the equilibrium state structure type such as in WYMV. In WYMV, there are two hairpins in IRES with an equilibrium-state structure, which is mediated by discontinuous C–G base pairing between the two hairpins: dynamic base pairs among cytosines (C^55^, C^66^, C^105^, and C^108^) in two hairpins and guanines (G^73^, G^79^, and G^85^) in the linker region. In addition to types I and II, there are several IRESes whose structural characteristics are unclear. The IRES in TuMV/PVA/PLRV has been classified as type III. Although there are three type of IRESes based on their structural characteristics, IRESes in plant RNA viruses appear to have simpler structure than that in animal RNA viruses. Similarly, IRESes of mammalian cellular mRNA are also less structured than that in animal RNA viruses [163,164]. In addition, IREses in yeast and fruit fly exhibit a weak secondary structure, which was correlated with high IRES activity [165]. With the exception of animal RNA viruses, RNAs from other resources including plant RNA viruses contain less structured IRESes. The less structured nature of IRESes in plant RNA viruses may be related to the shorter 5′UTR than that in animal RNA viruses. In addition, the 5′UTR length (a median length of approximately 53–218 nucleotides) of cellular mRNA appears to be shorter than that of IRESes (at least 450 nt) in animal RNA viruses [166]. A subset of eukaryotic IRESs exhibit very low secondary structure in the 5′UTR sequences immediately upstream of the initiation codon [165]. However, not all IRESes in plant RNA viruses exhibit a less structure characteristic. For instance, IRESes in WYMV presented a tertiary equilibrium-state structure, which was an alternative complex structure of IRES [103]. It has been suggested that the length of IRES sequences is not the sole determinant on the complexity of tertiary structure. The determinant of structure complexity of IRES can be a future research direction, in addition to the dialectic relationship between IRES structure and IRES activity.

In addition to the different structural characteristics, IRESes in plant RNA viruses present different mechanisms to recruit translation factors or ribosomes. Some IRESes such as TCV and TEV can bind eIF4G, while others such as WYMV can bind eIF4E. In addition, some IRESes, such as BRV, TEV and TriMV, can directly bind 18S rRNA. Although the genomic RNA of these plant RNA viruses does not contain the 5′cap, they can recruit the translation initiation complex by binding to specific components, such as eIF4E, eIF4G, and/or 18S rRNA. To date, IRESes of plant RNA viruses have appeared to function without the assistance of ITAF, while some IRESes in animal RNA viruses and cellular mRNA required ITAF [118,119,120,121,122,123,124,164,167,168]. Whether some potential ITAFs modulate the activity of IRESes in plant RNA viruses can be a future research direction. According to the sequence or structural characteristics of these IRESes in plant RNA viruses, a potential control strategy for the plant RNA viruses was tested. In studies of TuMV, TriMV and WYMV, complementary oligonucleotide with core *cis*-elements shows remarkable inhibition on translation [113,135], which implied that complementary oligonucleotides could be efficient agents against plant disease through the inhibition of translation of viral proteins. In addition, other types of small molecules, such as specific nucleotides and peptides, can be used as inhibitors of viral disease if they can block the essential interactions of IRESes and host translation factors or ribosomes. These types of molecules have been reported in studies of animal RNA viruses [169,170,171,172,173,174], which implies that this strategy can be applied to the control of diseases caused by plant RNA viruses. Small molecules blocking the core region in IRESes or the essential interaction between IRESes and host translation factors or ribosomes are potential agents for the management of viruses. In addition to resolving the detailed characteristic of core *cis*-elements in IRESes, the precise interaction sites between IRESes and host translation factors or ribosomes can be a future research direction, which will provide insight into the design of small-molecule blocking agents.

The primary function of IRES is the regulation of cap-independent translation, and different types of IRESes have been identified in some plant RNA viruses, which recruit translational initiation factors in different manners (Table 2). In addition to regulating cap-independent translation, the IRES of WYMV can play a positive role in regulating the translation of RNA with 5′ cap [113]. It implied a potential interaction between IRES and the 5′ cap in translation. Similarly, the 5′UTR (14 nt and 21 nt) of RNA3 and RNA10 in RBSDV presents IRES activity and can enhance the translation of RNA with a 5′cap [175]. The mechanism of the potential synergistic function between the 5′ cap and IRES requires further identification in future.

### 3.2. 3′CITE in Plant RNA Viruses

The 3’CITE was firstly reported in sTNV [104] and subsequently discovered in a large number of positive-strand RNA plant viruses [2,176,177]. 3’CITEs have also been found in both eukaryotic cells and animal RNA viruses [112,178,179]. In general, 3’CITEs can recruit diverse translation initiation factors or directly recruit and bind to the ribosome subunit, and the translation initiation complex is subsequently brought to the 5’ end of the RNA through long-distance RNA–RNA interaction to initiate translation [2]. Detailed characteristics of 3’CITEs have mainly been determined for plant RNA viruses. According to their recruitment on different host translation initiation factors, RNA structures, and circularization mechanisms, these 3’CITEs can be classified into seven classes: TED, BTE, PTE, TSS, ISS, YSS, and CXTE (Table 3).

#### 3.2.1. Translation Enhancer Domain (TED)

TED was discovered in the 3′UTR (619 nt) of sTNV, a parasitic subviral agent, and it enhances translation in vitro and in vivo [104]. Its structure is predicted to form a long hairpin containing several internal bulges and a 6 nt apical loop, which has no strong uninterrupted helices [104,180,198,199]. TED can recruit eIF4F or eIFiso4F, typically preferring eIF4F, which is essential for translation functions [200,201]. The apical loop of TED in sTNV contains sequences complemented by the apical loop of the 5′UTR, while mutation to disrupt the potential base pairing slightly reduces translation [180]. The detailed mechanism of how the ribosome complex recruited by TED is brought to the 5′UTR remains unclear.

Similar TED elements have been experimentally verified for other related viruses. Three carmoviruses including pelargonium line pattern virus (PLPV), pelargonium chlorotic ring pattern virus, and pelargonium ring spot virus have been shown to contain a TED-like element, which has sequences in its apical loops putatively forming a kissing-loop interaction with a 5′ proximal hairpin [187]. The core sequences (YGCCA; Y is a pyrimidine) in the apical loop of the TED-like element are conserved, which mediate the long-distance kissing-loop interaction with the 5′ proximal sequences. When the predicted long-range base pairing with the TED-like element in the PLPV is disrupted, the translation efficiency is reduced to less than 10% of the wt levels. It has been revealed that maintenance of the 5′–3′ gRNA communication is imperative for efficient translation mediated by the TED-like element [98].

#### 3.2.2. Barley Yellow Dwarf Virus (BYDV)-Like Element (BTE)

BTE was first reported in BYDV and subsequently found in all species of the genus *Luteovirus*, as well as in some species of the genera *Necrovirus*, *Umbravirus*, and *Dianthovirus* [182,202]. BTE contains a highly conserved 17 nt sequence (GGAUCCUGGNRNACAGG, the underlined base pair; N is any base and R is a purine) and a stable stem-loop SL-IIII pairing with the 5′UTR (140 nt) [183,203]. This long-distance RNA–RNA interaction between BTE and the 5′ ends of the gRNAs and/or sgRNAs is necessary for efficient translation [183]. eIF3 can bind to both the UTRs of BYDV to stabilize the 3′UTR–5′UTR interaction and facilitate the transfer of the translation machinery from the 3′BTE to the 5′UTR [204]. BTE of BYDV bound eIF4G with unusually high affinity and recruited translation machinery in an eIF4G-dependent manner [205]. The three-dimensional structure of BTE of BYDV was determined via crystallization and preliminary X-ray diffraction analysis [206].

BTE-like elements have also been reported in tobacco bushy top virus (TBTV) [207]. A study on the BTE of TBTV identified the structural evolution of BTE, which is mediated by the mutation of nucleotides outside of the BTE regions at the 3′ end. It is suggested that other regions at the 3′ end regulate translation by affecting the structure of the BTE region [207]. In addition, we found that the 5′ terminal region of the TBTV genome has a local molecule regulating the formation or deformation of long-distance RNA–RNA interactions between the 5′UTR (10 nt) and BTE (G. Geng and X. Yuan, unpublished data). In addition, a recent study revealed that opium poppy mosaic virus (OPMV) has a BTE at the 3′ end, which also contains another 3′CITE termed TSS [208]. The BTE, not the TSS in OPMV, contributes to the translation of the reporter constructs [208]. In addition to the conserved 17 nt sequences, BTE in species of the genus *Umbravirus* and seven additional BTEs from species of the family *Tombusviridae* and *Luteoviridae* have additional structural and sequence similarities, including the distance between SL1 and SL2, conserved sequences located downstream of SL2 and SL3 [208].

#### 3.2.3. Panicum Mosaic Virus-Like Translation Element (PTE)

PTE is present in several species of the genera *Carmovirus* and *Panicovirus*, pea enation mosaic virus RNA 2 (PEMV2) of the genus *Umbravirus*, and pothos latent virus of the genus *Aureusvirus* [187]. The PTE consists of a three-way branched helix with a large G-rich bulge (G domain) in the main stem and two helical branches at the branch point with a short C- or pyrimidine-rich bulge (C domain) [186]. The 5′ side hairpin of PTEs, excluding the PTE in PEMV2, has an apical loop complementary to the apical loop of a hairpin at or near the 5′ end of the viral RNA. In PEMV2, the translation complex recruited by the PTE is brought to the 5′ end through the 5′–3′ interaction mediated by kl-TSS. Meanwhile, the PTE of PEMV2 has been shown to bind eIF4E with high affinity [187,188]. MCMV also contains a 3′CITE mainly similar to PTE, termed MTE, which can interact with eIF4E with high affinity [209]. However, MTE lacks a strong pseudoknot, unlike most PTEs, and stimulates cap-independent translation with less efficiency than most PTEs [209].

#### 3.2.4. T-Shaped Structure (TSS)

TSS was first discovered in the turnip crinkle virus (TCV) of the genus *Carmovirus* [189]. The TSS contains a unique set of three hairpins and two pseudoknots that fold into a structure similar to that of tRNAs, as predicted by molecular modeling and confirmed by small-angle X-ray scattering (SAXS)/NMR, which was the first resolved 3D structure of a 3′CITE [190]. The TSS of TCV binds to the ribosome 60S subunit, and this binding is not only important for TSS activity, but also for circularization of the RNA template [189]. In addition, TSS is a scaffold that forms a highly interactive structure at the 3′ end of TCV, which undergoes a widespread conformation shift upon binding to RNA-dependent RNA polymerase [210,211].

A similar TSS structure has been proposed for the related cardamine chlorotic fleck virus of the genus *Carmovirus*. The 3′UTR of PEMV2 of the genus *Umbravirus* contains two functional TSSs. One is termed kl-TSS, located 9 nt upstream of the PTE, and the other is termed 3′TSS, located near the 3′ end of the genomic RNA, which is predicted to fold into structures similar to tRNAs [191,192]. In addition, TSS was also discovered in TBTV of the genus *Umbravirus* (X. Yuan, unpublished data) and also contained BTE, another type of 3′CITE [207].

#### 3.2.5. I-Shaped Structure (ISS)

ISS, the shortest 3′CITE, has been found in the different genera of the family *Tombusviridae* such as maize necrotic spot virus of the genus *Tombusvirus* and melon necrotic spot virus (MNSV) of the genus *Carmovirus* [194,212]. The ISS consists of a stem-loop structure (approximately 60 nt) with a four-base helix and flanking bulged sequences, which is similar to the RNA structure of TED. However, the sequences and motifs between the ISS and TED are fundamentally different. Two different types of ISS were previously identified in different MNSV isolates: MNSV-Ma5 and MNSV-264. Although there is considerable sequence divergence between the 3′CITEs of the MNSV isolates, all have the shape of an “I”. ISS binds the translation initiation factor eIF4F and engages in an RNA–RNA kissing-loop interaction with a hairpin loop located at the 5′ end of the genomic RNA [194,195,212].

#### 3.2.6. Y-Shaped Structure (YSS)

Nearly all members of the genus *Tombusvirus* are predicted to have a conserved YSS at the 3′ end [2,99,196]. The YSS consists of three long helices (SL-A, SL-B, and SL-C) protruding from a central hub and folded into structures similar to the shape of a “Y”. Mutations in three extended helices altering the structure of the stem or bulge formations reduce translation mediated by the YSS of TBSV [196]. The YSS of TBSV engages a 5′–3′ RNA–RNA interaction to facilitate cap- and poly(A)-independent translation [99]. YSS has also been discovered in carnation Italian ring spot virus and pelargonium leaf curl virus, whose activity for efficient translation requires eIF4F or eIFiso4F [213].

#### 3.2.7. Cucurbit Aphid-Borne Yellows Virus (CABYV) Xinjiang-Like Translation Element (CXTE)

A small 3′CITE termed CXTE was identified from the 3′UTR of the MNSV-N isolate, which can overcome eIF4E-mediated resistance due to the insertion of 55 nt sequences from CABYV [197]. These 55 nt sequences form a new 3′-CITE called CXTE. In the MNSV-N isolate, CXTE is responsible for recruiting translation machinery, and ISS is responsible for the formation of the 5′–3′ interaction [2,197]. Cap-independent translation mediated by CXTE may occur in an eIF4E-independent manner. This is the first report of 3′CITE transferring between different families by recombination in nature [197].

To date, the detailed characteristics of 3′CITE have been mainly identified in plant RNA viruses. Different 3′CITEs present different structural characteristics and regulate translation in a different manner recruiting host translation machinery (Table 3). Some plant RNA viruses such as species of *Umbravirus* contain multiple 3′CITEs. BTE, not TSS, in OPMV plays a role in cap-independent translation [208]. However, two types of 3′CITEs (CXTE and ISS) synergistically play a role in translation in the case of MNSV-N [197]. PTE and kl-TSS also synergistically play a role in translation in the case of PEMV 2 [192]. In addition to plant RNA viruses, there are many possible 3′CITEs in animal RNA viruses and eukaryotic cellular mRNAs [112,178,179], whose characterization is the virgin land for cap-independent translation and even translation regulation. This implies the universal existence of 3′CITE in different types of RNA, even in different organisms. Characteristics and mechanisms of 3′CITE in plant RNA viruses, animal RNA viruses, and eukaryotic cellular mRNA need to be identified, which will provide new insight into the translational regulation and evolution of different types of RNA. In addition, the 3′ end of genomic RNA in plant RNA viruses has also been reported to contain *cis*-elements regulating viral replication, which is another important biological process in viral life cycle. 3′CITE and replication-associated *cis*-elements located at the 3′end of virus genome may induce reciprocal actions or effects due to the local tertiary structure [210,211], which can be a future research direction for the deeper characterization of 3′CITE. In addition, switching between translation mediated by 3′CITE and replication mediated by corresponding *cis*-elements may also play an essential role in the viral life cycle, because the 3′ end could undergo a structural change upon binding to RdRp, the core component of viral replication [210].

**Figure 1:**
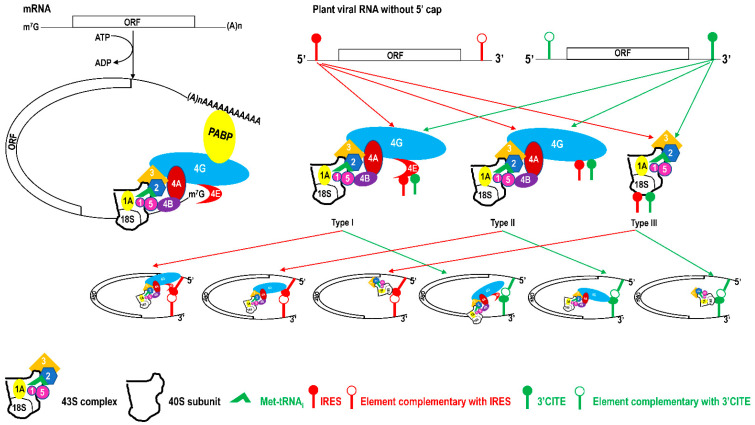
Schematics of translation initiation in cellular mRNA and plant viral RNA without 5′cap. All eukaryotic initiation factors (eIFs) are indicated by number as 1, 1A, 2, 3, 4A, 4B, 4E, 4G, and 5. PABP, poly(A)-binding protein.

IRES and 3′CITE are two types of *cis*-elements that mediate the cap-independent translation of viral RNA without the 5′ cap. According to information from previous studies and this review, IRES and 3′CITE in plant RNA viruses appear to present different structural characteristics. IRESes in plant RNA viruses present one of three characteristics: unstructured, structured, or unclear structure (Table 2). All 3′CITEs present seven types of remarkable structural characteristics (Table 3). Although the structure presents variety with few similarities, some conserved characteristics can be identified related to translation initiation factors or ribosome RNA bound by IRES or 3′CITE. According to the reported data, IRES or 3′CITE can bind to at least one of three components including eIF4E, eIF4G, or 18S RNA, which are important components for the translation initiation complex. It is suggested that both IRES and 3′CITE can recruit the translation initiation complex through three different pathways: type I, II, or III (Figure 1). Through the interaction with eIF4E, eIF4G, and/or 18S RNA, IRES or 3′CITE in plant RNA viruses can recruit the host translation initiation complex to ensure the translation initiation of viral RNA (Figure 1). In addition, cap-independent translation is also synergistically enhanced by viral RNA civilization mediated by the long-distance interaction between IRES or 3′CITE and *cis*-elements located at another end of the viral genome (Figure 1). *cis*-Elements in IRES and/or 3′CITE involved in the interaction with host translation initiation complex or the civilization of viral RNAs are the target for the design the small-molecule agents for the management of virus diseases.

## 4. Translation Recoding

Cap-independent translation is responsible for the expression of viral proteins located at the 5′ proximal region in viral genomic RNAs or subgenomic RNAs without the 5′ cap. However, some plant RNA viruses are multi-cistronic, and viral proteins located at the internal or 3′ part of genomic RNAs are expressed through other strategies at the transcriptional or translational level. At the transcriptional level, some plant RNA viruses can produce subgenomic RNAs, in which some viral proteins located at the internal or 3′ part of the genomic RNAs are changed to 5′ proximal [1]. At the translational level, these proteins unlocated at the 5′ proximal in genomic or subgenomic RNAs can be expressed through translational recoding, which is an alternative method of identifying the message of the initiation or stop codon of the ORF. Translation recoding includes several types: leaky scanning or non-AUG initiation standing for the recoding of the initiation codon, ribosomal read-through or ribosomal frameshift standing for the recoding of the stop codon, and translational bypassing standing for special expression of the peptide from a discontinuous frame [3,214,215].

### 4.1. Leaky Scanning

Leaky scanning was first discovered in the genus *Orthobunyavirus*, in which the ORF of NSs was located completely inside the ORF of N. The NSs are expressed via leaky scanning at the AUG of the ORF of the N protein [216]. Leaky scanning is a mechanism of the translation initiation complex skipping the first initiation codon AUG of the corresponding ORF and initiating translation at the downstream initiation codon AUG due to the nonoptimal context surrounding the first initiation codon AUG [217]. The optical context of the initiation codon AUG is (A/G)CCAUGG in mammalian systems and ACAAUGG in the plant systems, in which purine (A/G) at the −3 position and G at the +4 position are the strongest indicators of translation initiation in animals, plants, and fungi [218,219,220]. This process allows the expression of multiple C-terminally coincident isoforms of a single protein (in-frame alternative initiation sites), distinct proteins encoded by different overlapping ORFs (alternative initiation sites in different reading frames), or even distinct proteins encoded by nonoverlapping continuous ORFs [221].

According to the genome organization of plant RNA viruses, leaky scanning can occur at eight genera in the family *Alphaflexiviridae*, three genera in the family *Betaflexivridae*, one genus in the family *Benyviridae*, one genus in the family *Kitaviridae*, three genera in the family *Luteoviridae*, two genera in the family *Reoviridae*, three genera in the family *Secoviridae*, two genera in the family *Solemoviridae*, six genera in the family *Virgaviridae*, nine genera in the family *Tombusviridae*, and three genera in the family *Tymoviridae* (Table 1). Leaky scanning is a universal translation recoding strategy for plant RNA viruses. The 17K protein in BYDV-PAV sgRNA1 is produced by leaky scanning from the start codon (UGA**AUG**A) of CP ORF, in which the leaky ratio was about 50% [222]. The p39 protein is the leaky scanning product of p23 in the peanut clump virus, in which the ratio of leaky scanning on p23 was 20–30% [223]. βc protein is translated using a leaky scanning mechanism from the start codon ofβd ORF in RNAβ of barley strip mosaic virus [224]. Rice tungro bacilliform virus (RTBV) can express the internal ORFs II and III by leaky scanning [225]. In potato virus X, the 8K ORF is translated by leaky ribosome scanning through the 12K ORF [226]. In the PLPV, the expression of p9.7 is the leaky scanning product from p7 with the ratio of 10% of p7. In addition, p37 is produced through the leaky scanning over the start codon of p7 and non-AUG start codon of p9.7, and the ratio of p37 to p7 was about 50% [227]. Translation of ORF 2a in sobemoviruses is also dependent on the leaky scanning mechanism [228].

### 4.2. Non-AUG Initiation

The leaky scanning mechanism implies the importance of the context of initiation codon AUG, which can also be confirmed by another translation recoding mechanism termed non-AUG initiation. No AUG initiation was first discovered in the sendai virus in 1988, and translation can be initiated from the ACG codon [229]. In the non-AUG initiation strategy, some codons such as CUG, GUG, ACG, AUU, AUA, AUC, and UUG have been confirmed to initiate translation at a 2–30% level, and CUG surrounded by an optimal context is the most efficient non-AUG initiation codon [230]. Initiation at a non-AUG codon normally requires a strict context such as an A or G at −3 and a G at +4. In addition, a stem loop located at approximately 14 nt downstream of the initiation codon could enhance non-AUG initiation [231,232]. Non-AUG initiation has been discovered in many plant viruses, such as RTBV, species of the family *Tombuviridae*, and species of the family *Panicovirus* [225,227,233,234].

### 4.3. Ribosomal Frameshift

During the special translation process, ribosomes show an abnormal shift of non-three codons on the RNA template, which changes the reading frame of the ORF. This phenomenon is termed ribosomal frameshift [235]. The ribosome can slip one or two nucleotides (−1 or −2 frameshifts) to the 5′ end or one or two nucleotides (+1 or +2 frameshifts) to the 3′ end [25,236,237,238], in which the programmed −1 ribosomal frameshift is the type with detailed characteristics [239]. Programmed −1 ribosomal frameshift was first described by the expression of the Gag–Pol polyprotein of the rous sarcoma alpharetrovirus, which is a chimeric product of overlapping gag and pol ORFs [240,241]. The −1 ribosomal frameshift is involved in several levels of *cis*-elements: a “slippery site” composed of seven nucleotides with the characteristic XXXYYYZ (X is any base, Y is A or U, and Z is not G) [241,242,243]; a downstream stimulatory structure, typically a stem loop or pseudoknot [244,245,246]; a suitable 5–9 nt between the slippery site and downstream stimulatory structure; an element at the 3′ end forming a long-distance RNA–RNA interaction with the downstream stimulatory structure [247,248,249].

Ribosomal frameshifting is an important gene expression strategy in plant RNA viruses, which can occur in one genus of the family *Amalgaviridae*, one genus of the family *Aspiviridae*, four genera of the family *Closteroviridae*, four genera of the family *Luteoviridae*, and two genera of the family *Solemoviridae* (Table 1). The ORF2a–ORF2b protein in PLRV, CP-12K protein in potato virus M, and the Pipo protein in TuMV are expressed via a −1 ribosomal frameshift [250,251]. PEMV2 expresses its RNA polymerase using a −1 ribosomal frameshift, which is regulated through multiple *cis*-acting elements [252]. The viral RdRp of species of the genus *Closterovirus* such as CTV and BYV is possibly translated through a +1 frameshift [253]. Viruses of the family *Luteoviridae* express ORF 2 via a −1 ribosomal frameshift from ORF 1, thereby giving an ORF 1/2 fusion protein [254]. p98 (RdRP) in TBTV is expressed via a −1 ribosomal frameshift [255], which is regulated by downstream kissing-linker and multiple pairs of long-distance RNA–RNA interactions between downstream regions of slippery sequences and the 3′ end (Yu and Yuan, unpublished data).

In addition to the above translational ribosomal frameshift, there is a transcriptional frameshift due to RNA editing on the RNA template to change the sequences of the ORF. The expression of P3N-PIPO in PVY and TuMV, as well as of P1N-PISPO in sweet potato feather mottle virus, has been confirmed through the transcriptional frameshift mechanism [251,256,257,258,259]. The transcriptional frameshift mechanism has also been found in prokaryotes, eukaryotes, and chloroplasts [260,261,262,263,264].

### 4.4. Ribosomal Read-Through

During the special translation process, the ribosome can fail to terminate translation at the stop codon and pass through the stop codon to produce a C-terminal extended peptide at a proportion of 0.3–5%. This phenomenon during translation is termed ribosomal read-through [265,266]. Ribosomal read-through was first discovered in TMV, in which read-through of an amber stop codon produced a 183K protein, which requires the existence of two naturally tyrosine-specific suppressor tRNAs [267,268,269]. Ribosomal read-through may be involved in several *cis*-elements including suppressor tRNA and a local stimulatory structure, such as a stem loop or pseudoknot, downstream of the stop codon, which is an element at the proximal 3′ end engaging in long-distance RNA–RNA interaction with the local stimulatory structure [270,271,272,273,274,275].

In plant RNA viruses, ribosomal read-through can occur in the genus *Benyvirus* in the family *Benyviridae*, three genera in the family *Luteoviridae*, seven genera in the family *Virgaviridae*, and 14 genera in the family *Tombusviridae* (Table 1) and is used to express two types of proteins: including replicase and CP [271]. The RNA replicase of TMV is translated by read-through [276]. In the genus *Tobamovirus*, the read-through replicase p182 is sufficient for viral replication and transcription [277]. The CP of BYDV is expressed by readthrough [278]. Tobacco necrosis virus-D expresses its polymerase via read-through [279]. The family *Luteoviridae* encodes two forms of CP: the major component, CP, and read-through protein (CP readthrough domain (RTD)) [280]. The readthrough (RT) product of CP is involved in vector transmission through specific interactions between CP-readthrough and vector proteins [270,281,282]. The ratio of CP-RT to CP is regulated such that the surface of the virion contains a suitable CP-RT to facilitate vector transmission. Replicases in the family *Tombusviridae* and *Virgaviridae* are expressed via read-through; the ratio of read-through is about 5–10% and is regulated by several *cis*-elements [283]. During genome replication, plant RNA viruses produce dsRNA, which can induce gene-silencing cleavage. These plant RNA viruses express replicase at a suitable level via read-through to control the speed of replication of the genome.

Although different types of translation recoding in plant RNA viruses present different mechanisms involving different *cis*-elements and various viral or host *trans*-factors, the expression of translation recoding products at the suitable level is the common point for different RNA viruses. The suitable ratio of translation recoding is essential for fitness of corresponding RNA viruses, suggesting that small molecules interfering with the suitable ratio of translation recoding in plant RNA viruses can be effective agents for the management of virus diseases. The design of this type of small molecule relies on the detailed characterization of *cis*-elements or *trans*-factors involved in the different translation recoding processes, which is also a future research direction in translation recoding.

## 5. Conclusions

IRESes and 3’CITEes in plant RNA viruses presented different structure characteristic in different viruses and recruited host translation machinery through eIF4E, eIF4G or 18s rRNA. However, detailed information about interaction between cap-independent translation elements and translation machinery was rough, which require further identification in future. Both ribosomal frameshift and ribosomal read through in plant RNA viruses were involved in several levels of cis-elements, but detailed characterization on structure of these core cis-elements was few and rough. In addition, potential relationship between the ratio of translation recoding and virus fitness was unclear. During translation regulation of plant RNA viruses, different RNA cis-element may undergo structure shift on the interaction with other cis- or- trans- element and/or factors. However, the study on the structure shift of *cis*-elements was few. All these information gap will be future direction.

Translation of viral proteins is a vital process during the life cycle of viruses. Comprehensive and persistent identification of different translation strategies in plant RNA viruses will provide new insights into translation regulation and new mechanisms in virus evolution, which will result in new strategies, target sites, and agents for the management of viral diseases of plants.

## Figures and Tables

**Table 1:** Characteristic of 5′ and 3′ ends of plant RNA viruses and their potential translational recoding strategy.

Order	Family	Subfamily	Genus	Viral RNA or Subgenomic RNA		Translation Recoding Strategy	References
				5′ End	3′ End		
Bunyavirales	*Fimoviridae*		*Emaravirus*	5′ cap	no 3′ poly(A)	/	[8]
	*Phenuiviridae*		*Coguvirus*	5′ cap	no 3′ poly(A)	/	[9]
			*Rubodvirus*	5′ cap	no 3′ poly(A)	/	[10]
			*Tenuivirus*	5′ cap	no 3′ poly(A)	/	[11]
	*Tospoviridae*		*Orthotospovirus*	5′ cap	no 3′ poly(A)	/	[12]
Durnavirales	*Amalgaviridae*		*Amalgavirus*	no 5′ cap	no 3′ poly(A)	ribosomal frameshift	[13]
	*Partitiviridae*		*Alphapartitivirus*	no 5′ cap	3′ poly(A)	/	[14]
			*Betapartitivirus*	no 5′ cap	3′ poly(A)	/	[15]
			*Deltapartitivirus*	no 5′ cap	3′ poly(A)	/	[16]
Hepelivirales	*Benyviridae*		*Benyvirus*	5’ cap	3′ poly(A)	ribosomal read-through, leaky scanning	[17]
Martellivirales	*Bromoviridae*		*Alfamovirus*	5’ cap	no 3′ poly(A), has a 3’ tRNA-like structure	/	[18]
			*Anulavirus*	5’ cap	no 3′ poly(A), has a 3’ tRNA-like structure	/	[19]
			*Bromovirus*	5’ cap	no 3′ poly(A), has a 3’ tRNA-like structure	/	[20]
			*Cucumovirus*	5’ cap	no 3′ poly(A), has a 3’ tRNA-like structure	/	[21]
			*Ilarvirus*	5’ cap	no 3′ poly(A), has a 3’ tRNA-like structure	/	[22]
			*Oleavirus*	5’ cap	no 3′ poly(A), has a 3’ tRNA-like structure	/	[23]
	*Closteroviridae*		*Ampelovirus*	5′ cap	no 3′ poly(A)	ribosomal frameshift	[24]
			*Closterovirus*	5′ cap	no 3′ poly(A)	ribosomal frameshift	[25]
			*Crinivirus*	5′ cap	no 3′ poly(A)	ribosomal frameshift	[26]
			*Velarivirus*	5′ cap	no 3′ poly(A)	ribosomal frameshift	[27]
	*Endornaviridae*		*Alphaendornavirus*	no 5′ cap	no 3′ poly(A)	/	[28]
	*Kitaviridae*		*Blunervirus*	5′ cap	no 3′ poly(A)	leaky scanning	[29]
			*Cilevirus*	5′ cap	3′ poly(A)	/	[30]
			*Higrevirus*	5′ cap	3′ poly(A)	/	[31]
	*Mayoviridae*		*Idaeovirus*	5′ cap	no 3′ poly(A)	/	[32]
			*Pteridovirus*	5′ cap	no 3′ poly(A)	/	[33]
	*Virgaviridae*		*Furovirus*	5′ cap	no 3′ poly(A), has a 3′ tRNA-like structure	ribosomal read-through, leaky scanning	[34]
			*Goravirus*	5′ cap	no 3′ poly(A), has a 3′ tRNA-like structure	ribosomal read-through, leaky scanning	[35]
			*Hordeivirus*	5′ cap	no 3′ poly(A), has a 3′ tRNA-like structure	ribosomal read-through, leaky scanning	[36]
			*Pecluvirus*	5′ cap	no 3′ poly(A), has a 3′ tRNA-like structure	ribosomal read-through, leaky scanning	[37]
			*Pomovirus*	5′ cap	no 3′ poly(A), has a 3′ tRNA-like structure	ribosomal read-through, leaky scanning	[38]
			*Tobamovirus*	5′ cap	no 3′ poly(A), has a 3′ tRNA-like structure	ribosomal read-through	[39]
			*Tobravirus*	5′ cap	no 3′ poly(A), has a 3′ tRNA-like structure	ribosomal read-through, leaky scanning	[40]
Mononegavirales	*Rhabdoviridae*		*Alphanucleorhabdovirus*	5′ cap	3′ poly(A)	/	[41]
			*Betanucleorhabdovirus*	5′ cap	3′ poly(A)	/	[42]
			*Gammanucleorhabdovirus*	5′ cap	3′ poly(A)	/	[43]
			*Cytorhabdovirus*	5′ cap	3′ poly(A)	/	[44]
			*Dichorhavirus*	5′ cap	3′ poly(A)	/	[45]
			*Varicosavirus*	5′ cap	3′ poly(A)	/	[46]
Ourlivirales	*Botourmiaviridae*		*Ourmiavirus*	no 5′ cap	no 3′ poly(A)	/	[47]
Patatavirales	*Potyviridae*		*Arepavirus*	5’ VPg	3′ poly(A)	/	[48]
			*Bevemovirus*	5’ VPg	3′ poly(A)	/	[49]
			*Brambyvirus*	5’ VPg	3′ poly(A)	/	[50]
			*Bymovirus*	5’ VPg	3′ poly(A)	/	[51]
			*Celavirus*	5’ VPg	3′ poly(A)	/	[52]
			*Ipomovirus*	5’ VPg	3′ poly(A)	/	[53]
			*Macluravirus*	5’ VPg	3′ poly(A)	/	[54]
			*Poacevirus*	5’ VPg	3′ poly(A)	/	[55]
			*Potyvirus*	5’ VPg	3′ poly(A)	/	[56]
			*Roymovirus*	5’ VPg	3′ poly(A)	/	[57]
			*Rymovirus*	5’ VPg	3′ poly(A)	/	[58]
			*Tritimovirus*	5’ VPg	3′ poly(A)	/	[59]
Picornavirales	*Secoviridae*		*Cheravirus*	5’ VPg	3′ poly(A)	leaky scanning	[60]
			*Sadwavirus*	5’ VPg	3′ poly(A)	/	[61]
			*Sequivirus*	5’ VPg	3′ poly(A)	/	[62]
			*Torradovirus*	5’ VPg	3′ poly(A)	leaky scanning	[61]
			*Waikavirus*	5’ VPg	3′ poly(A)	/	[62]
		*Comovirinae*	*Comovirus*	5’ VPg	3′ poly(A)	/	[61]
			*Fabavirus*	5’ VPg	3′ poly(A)	/	[61]
			*Nepovirus*	5’ VPg	3′ poly(A)	leaky scanning	[62,63]
Reovirales	*Reoviridae*	*Sedoreovirinae*	*Phytoreovirus*	5′ cap	no 3′ poly(A)	leaky scanning	[64]
		*Spinareovirinae*	*Fijivirus*	5′ cap	no 3′ poly(A)	/	[64]
			*Oryzavirus*	5′ cap	no 3′ poly(A)	leaky scanning	[64]
Serpentovirales	*Aspiviridae*		*Ophiovirus*	5′cap	no 3′ poly(A)	ribosomal frameshift	[65]
Sobelivirales	*Solemoviridae*		*Polemovirus*	5’ VPg	no 3′ poly(A)	ribosomal frameshift, leaky scanning	[66]
			*Sobemovirus*	5’ VPg	no 3′ poly(A)	ribosomal frameshift, leaky scanning	[67]
Tymovirales	*Alphaflexiviridae*		*Allexivirus*	no 5′ cap	3′ poly(A)	leaky scanning	[68]
			*Lolavirus*	no 5′ cap	3′ poly(A)	leaky scanning	[69]
			*Mandarivirus*	no 5′ cap	3′ poly(A)	leaky scanning	[69]
			*Platypuvirus*	no 5′ cap	3′ poly(A)	leaky scanning	[70]
			*Potexvirus*	5′ cap	3′ poly(A)	leaky scanning	[71]
		*Quinvirinae*	*Carlavirus*	5′ cap	3′ poly(A)	leaky scanning	[69,72]
			*Foveavirus*	5′ cap	3′ poly(A)	leaky scanning	[69,72,73]
			*Robigovirus*	5′ cap	3′ poly(A)	leaky scanning	[72]
	*Betaflexivridae*	*Trivirinae*	*Capillovirus*	no 5′ cap	3′ poly(A)	/	[73]
			*Chordovirus*	no 5′ cap	3′ poly(A)	/	[74]
			*Citrivirus*	no 5′ cap	3′ poly(A)	/	[75]
			*Divavirus*	no 5′ cap	3′ poly(A)	/	[76]
			*Prunevirus*	5′ cap	3′ poly(A)	leaky scanning	[77]
			*Ravavirus*	5′ cap	3′ poly(A)	leaky scanning	[77]
			*Tepovirus*	no 5′ cap	3′ poly(A)	/	[78]
			*Trichovirus*	no 5′ cap	3′ poly(A)	/	[73]
			*Vitivirus*	5′ cap	3′ poly(A)	leaky scanning	[73]
			*Wamavirus*	no 5′ cap	3′ poly(A)	leaky scanning	[79]
	*Luteoviridae*		*Enamovirus*	5′ VPg	no 3′ poly(A)	leaky scanning, ribosomal frameshift, ribosomal read-through	[80]
			*Luteovirus*	5′ VPg	no 3′ poly(A)	leaky scanning, ribosomal frameshift, ribosomal read-through	[81]
			*Polerovirus*	5′ VPg	no 3′ poly(A)	leaky scanning, ribosomal frameshift, ribosomal read-through	[82]
	*Tymoviridae*		*Maculavirus*	5′ cap	3′ poly(A)	leaky scanning	[83]
			*Marafivirus*	5′ cap	3′ poly(A)	leaky scanning	[84]
			*Tymovirus*	5′ cap	no 3′ poly(A), has a 3′ tRNA-like structure	leaky scanning	[85,86]
Tolivirales	*Tombusviridae*	*Calvusvirinae*	*Umbravirus*	no 5′ cap	no 3′ poly(A)	ribosomal frameshifting	[87]
		*Procedovirinae*	*Alphanecrovirus*	no 5′ cap	no 3′ poly(A)	ribosomal read-through	[88]
			*Alphacarmovirus*	no 5′ cap	no 3′ poly(A)	ribosomal read-through, leaky scanning	[89]
			*Aureusvirus*	no 5′ cap	no 3′ poly(A)	ribosomal read-through, leaking scanning	[90]
			*Avenavirus*	no 5′ cap	no 3′ poly(A)	ribosomal read-through	[91]
			*Betacarmovirus*	no 5′ cap	no 3′ poly(A)	ribosomal read-through, leaking scanning	[92]
			*Betanecrovirus*	no 5′ cap	no 3′ poly(A)	ribosomal read-through	[88]
			*Gallantivirus*	no 5′ cap	no 3′ poly(A)	ribosomal read-through	[93]
			*Gammacarmovirus*	no 5′ cap	no 3′ poly(A)	ribosomal read-through, leaking scanning	[94]
			*Macanavirus*	no 5′ cap	no 3′ poly(A)	ribosomal read-through	[95]
			*Machlomovirus*	no 5′ cap	no 3′ poly(A)	ribosomal read-through, leaking scanning	[96]
			*Panicovirus*	no 5′ cap	no 3′ poly(A)	ribosomal read-through, leaking scanning	[97]
			*Pelarspovirus*	no 5′ cap	no 3′ poly(A)	ribosomal read-through, leaking scanning	[98]
			*Tombusvirus*	no 5′ cap	no 3′ poly(A)	ribosomal read-through, leaking scanning	[99]
			*Zeavirus*	no 5′ cap	no 3′ poly(A)	ribosomal read-through, leaking scanning	[100]
		*Regressovirinae*	*Dianthovirus*	no 5′ cap	no 3′ poly(A)	ribosomal frameshift	[101]

Note: “/” indicating no translation recoding strategy based on genome organization and/or corresponding publication.

**Table 2:** Summary of IRESes in plant RNA viruses.

IRES Type	Group I with Unstructured *cis*-Elements		Group II with Structured *cis*-Elements				Group III with Unclear Structure Characteristic	
			Single-Stem Loop Structure	Double-Stem Loop Structure		Equilibrium-State Structure		
Virus	TCV/HCRSV/PFBV/crTMV	BRV	TEV	PVY	TriMV	WYMV	TuMV/PVA	PLRV
Structural characteristic	US-1	US-2	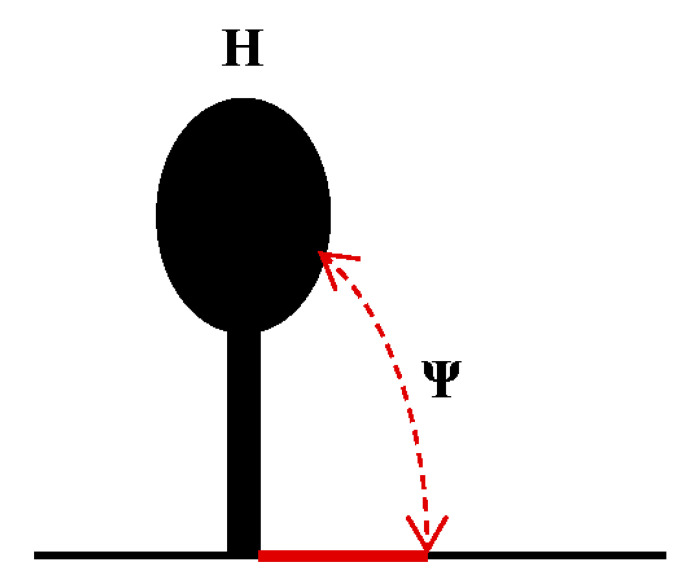	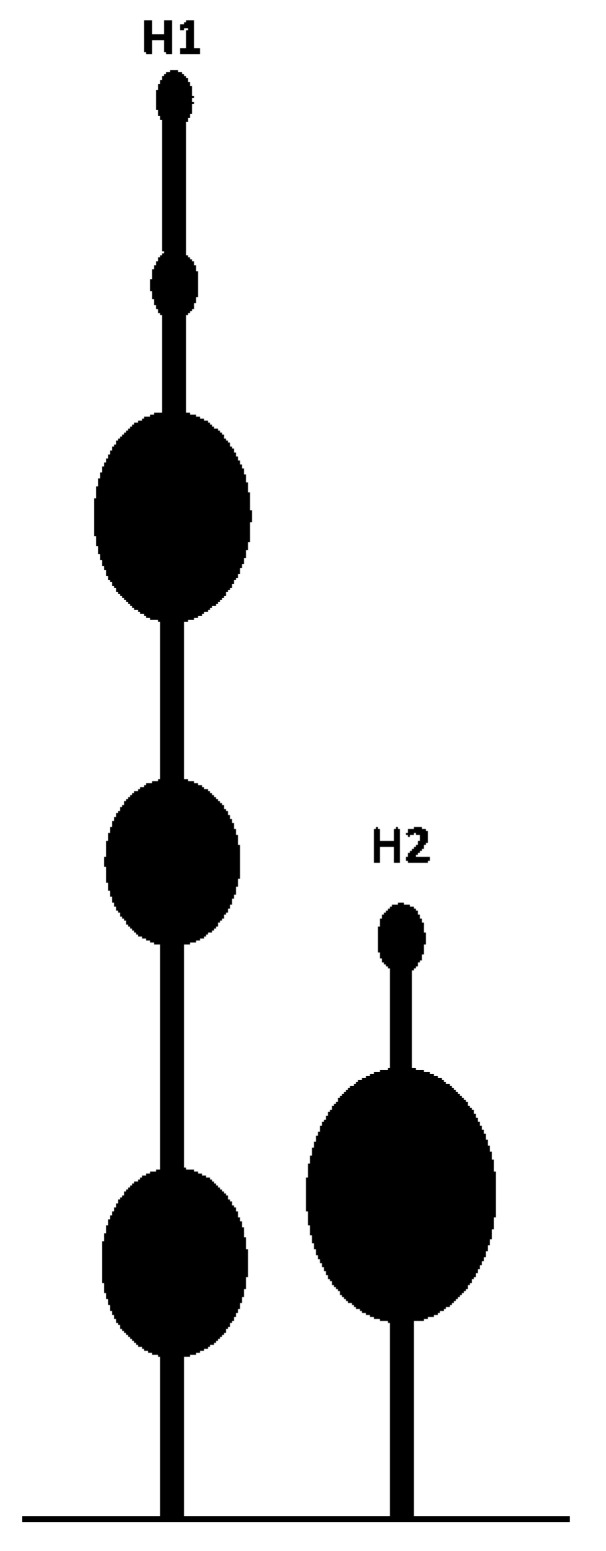	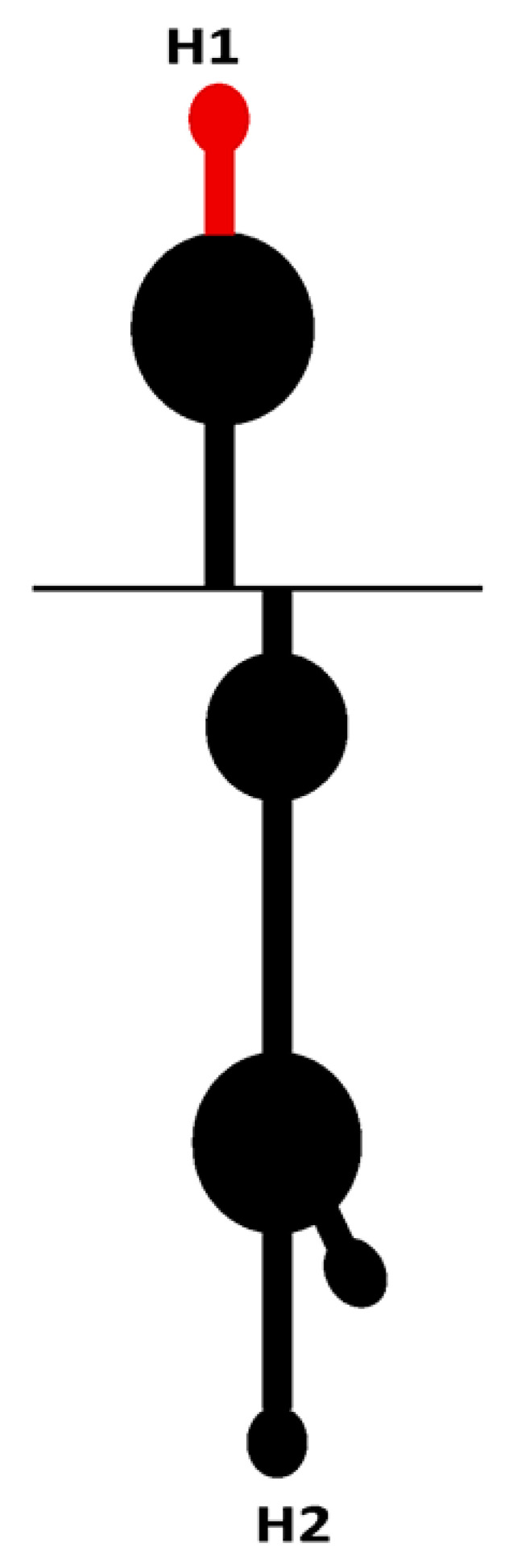	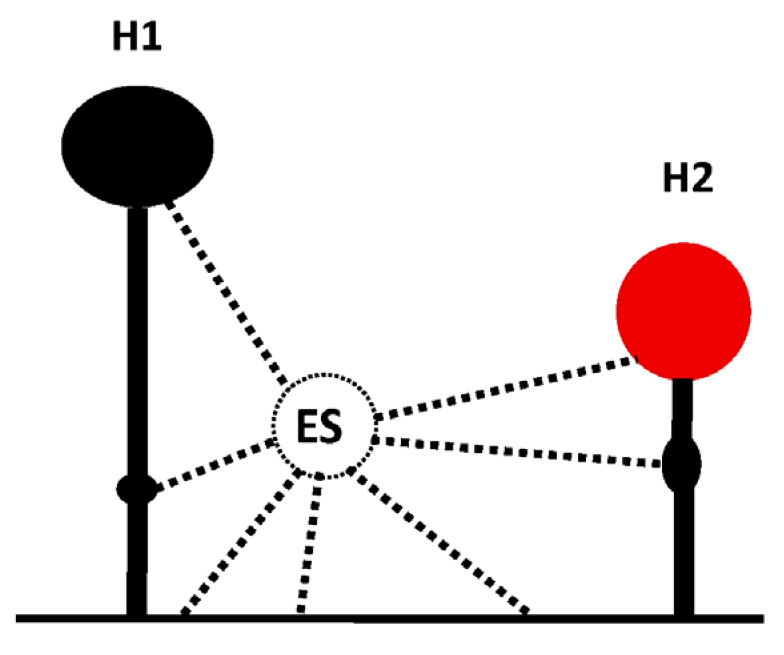	N/A	US-3
Binding translation factors	eIF4G	18S rRNA	eIF4G/eIF4F/18S rRNA	N/A	eIF4G/eIFis-o4G/eIF4A	eIF4E	N/A	N/A
References	[157,158,159,160]	[161]	[149,151]	[152]	[136,155]	[113]	[135,156]	[162]

Note: TCV, turnip crinkle virus; HCRSV, hibiscus chlorotic ringspot virus; PFBV, pelargonium flower break virus; crTMV, crucifer-infecting tobamovirus; BRV, blackcurrant reversion virus; TEV, tobacco etch virus; PVY, potato virus Y; TriMV, triticum mosaic virus; WYMV, wheat yellow mosaic virus; TuMV, turnip mosaic potyvirus; PLRV, potato leafroll polerovirus; ES, equilibrium state. N/A, not analyzed. Red part in RNA structures indicates the RNA *cis*-elements bound by corresponding translation initiation factors. US-1, a stretch of A-rich unstructured bases; US-2, six discontinuous motifs; US-3, a conserved AUG codon and a reverse symmetric downstream motif.

**Table 3:** Summary of 3′ CITEes in plant RNA viruses.

3′ CITE Type	TED	BTE	PTE	TSS	ISS	YSS	CXTE
Viruses	sTNV/PLPV/PCRPV	BYDV/TBTV	PMV/PEMV2	TCV/CCFV/ PEMV2	MNSV	TBSV/CIRV/ PLCV	CABYV
Structure	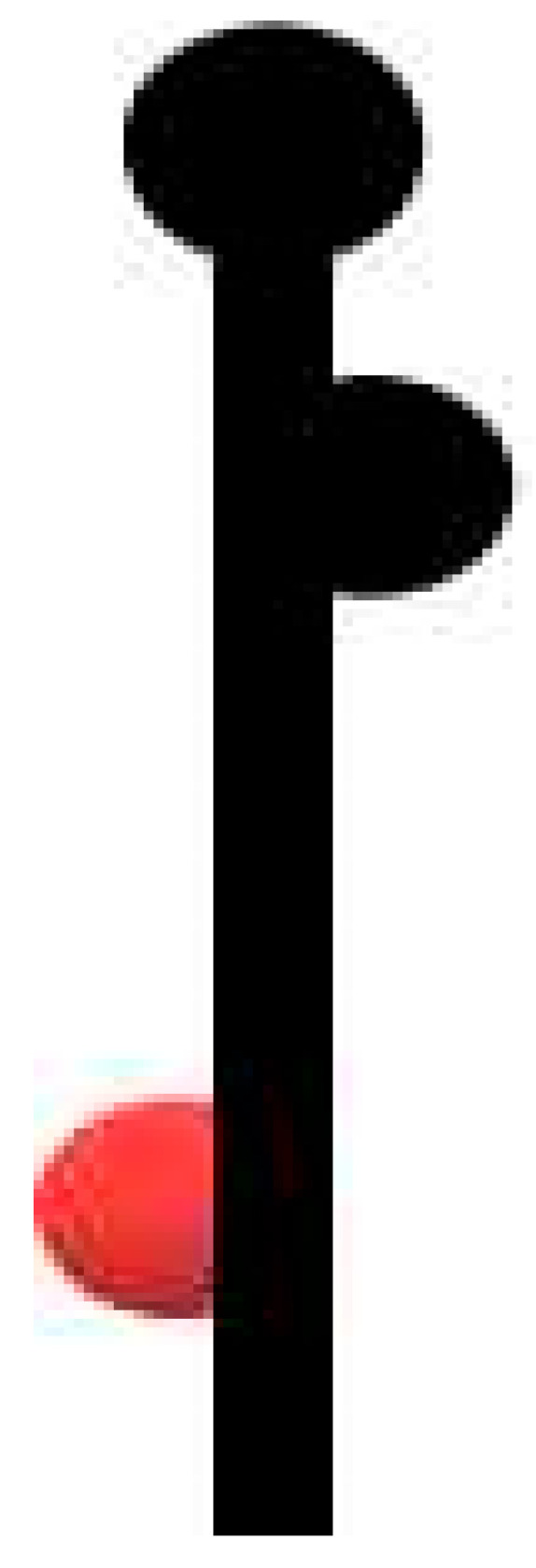	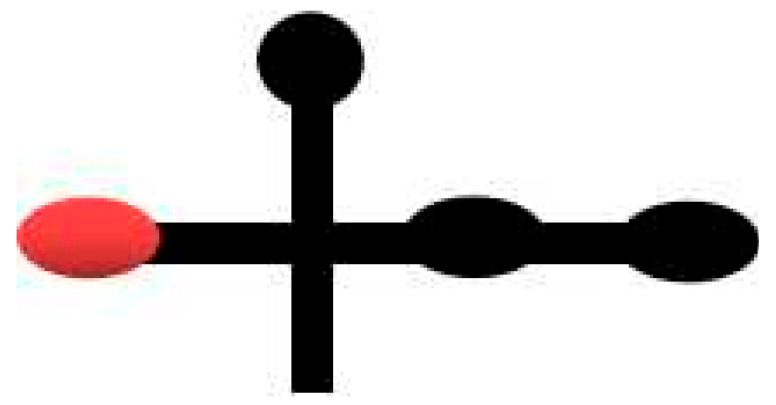	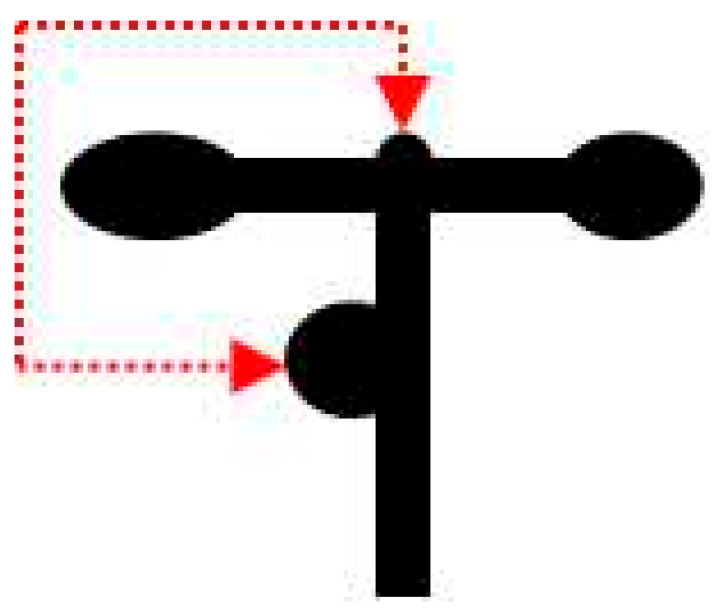	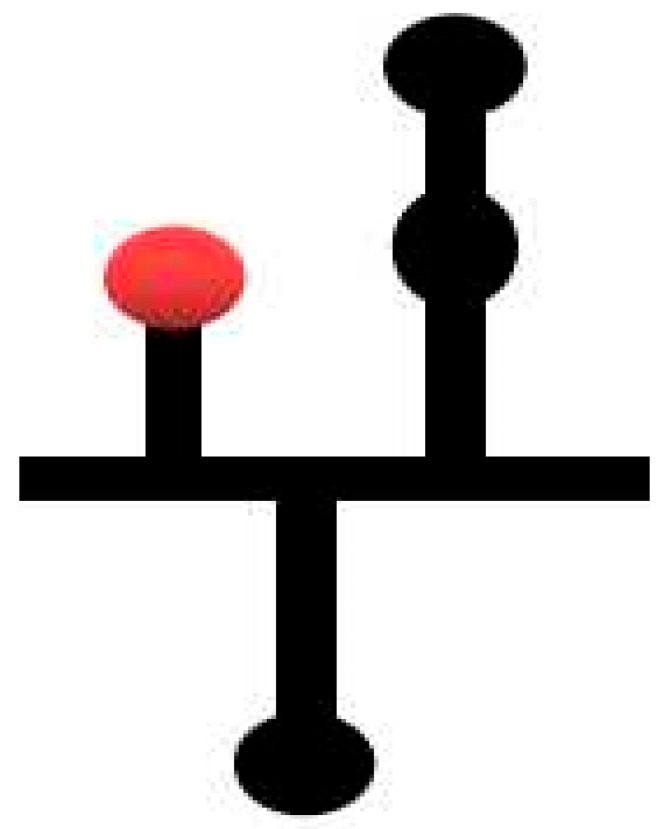	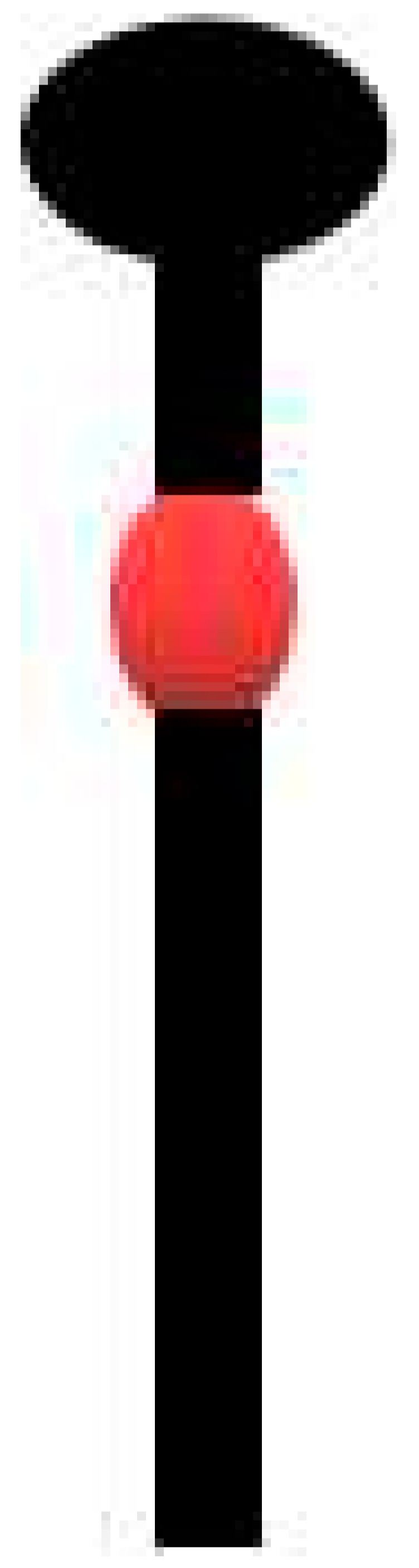	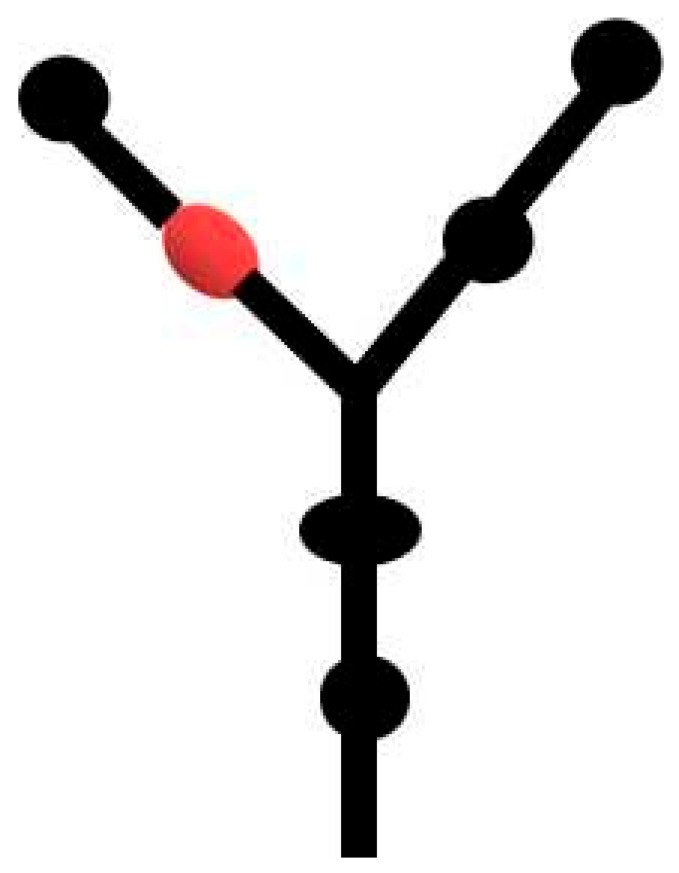	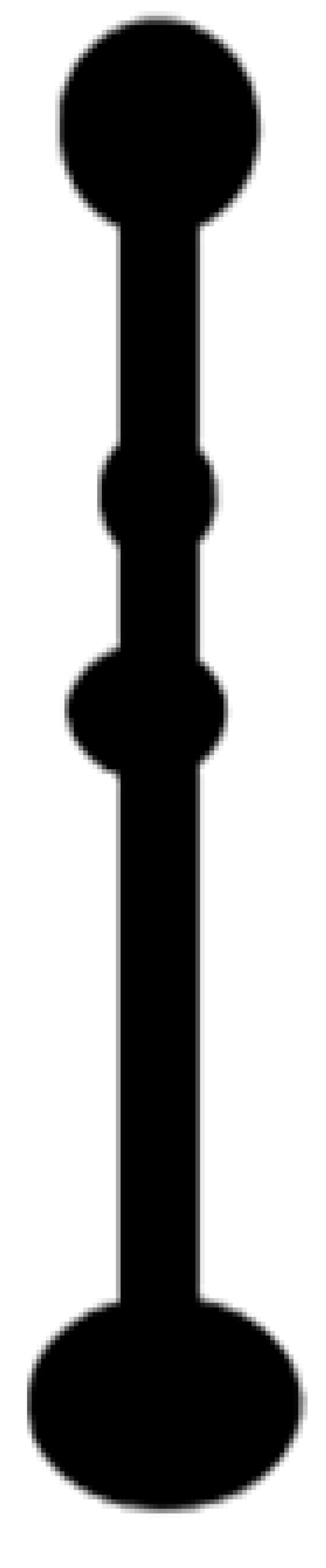
Binding translation factor	eIF4F/eIFiso4F	eIF4G	eIF4G/eIF4E	18S rRNA	eIF4G/eIF4F	eIF4F/eIFiso4F	N/A
References	[98,104,180,181]	[182,183,184]	[185,186,187,188]	[189,190,191,192]	[193,194,195]	[2,196]	[197]

Note: sTNV, satellite tobacco necrosis virus; PLPV, pelargonium line pattern virus; PCRPV, pelargonium chlorotic ring pattern virus; BYDV, barley yellow dwarf virus; TBTV, tobacco bushy top virus; PEMV2, pea enation mosaic virus RNA 2; TCV, turnip crinkle virus; CCFV, cardamine chlorotic fleck virus; CABYV, cucurbit aphid-borne yellows virus; TBSV, tomato bushy stunt virus; CIRV, carnation Italian ringspot virus; PLCV, pelargonium leaf curl virus; MNSV, melon necrotic spot virus; N/A, not analyzed. Red part in RNA structures indicates the RNA *cis*-elements bound by corresponding translation initiation factors.

## Data Availability

Not applicable.
